# Phospholipid-Conjugated PEG-*b*-PCL Copolymers as Precursors of Micellar Vehicles for Amphotericin B

**DOI:** 10.3390/polym13111747

**Published:** 2021-05-27

**Authors:** Elsa R. Arias, Vivian Angarita-Villamizar, Yolima Baena, Claudia Parra-Giraldo, Leon D. Perez

**Affiliations:** 1 Departamento de Química, Facultad de Ciencias, Universidad Nacional de Colombia-Sede Bogotá, Carrera 45 N° 26-85, Bogotá 11001, Colombia; erarias@unal.edu.co (E.R.A.); aeangaritav@unal.edu.co (V.A.-V.); 2 Grupo de Investigación SILICOMOBA, Departamento de Farmacia, Facultad de Ciencias, Universidad Nacional de Colombia-Sede Bogotá, Carrera 30 # 45-03, Bogotá 11001, Colombia; 3 Unidad de Proteómica y Micosis Humanas, Grupo de Enfermedades Infecciosas, Departamento de Microbiología, Facultad de Ciencias, Pontificia Universidad Javeriana, Bogotá 110231, Colombia

**Keywords:** amphotericin B, polymer micelle, phospholipid-modified copolymer

## Abstract

Amphotericin B (AmB) is a widely used antifungal that presents a broad action spectrum and few reports on the development of resistance. However, AmB is highly toxic, causing renal failure in a considerable number of treated patients. Although when AmB is transported via polymer micelles (PMs) as delivery vehicles its nephrotoxicity has been successfully attenuated, this type of nanoparticle has limitations, such as low encapsulation capacity and poor stability in aqueous media. In this research, the effect of modifying polyethyleglicol-*block*-poly(ε-caprolactone) (PEG-*b*-PCL) with 1,2-distearoyl-sn-glycero-3-phosphorylethanolamine (DSPE) on the performance of PMs as vehicles for AmB was studied. PEG-*b*-PCL with two different lengths of a PCL segment was prepared via ring opening polymerisation and modified with DSPE at a post-synthesis stage through amidation. Upon modification with DSPE, a copolymer was self-assembled, thereby producing particles with hydrodynamic diameters below 100 nm and a lower critical micelle concentration than that of the raw copolymers. Likewise, in the presence of DSPE, the loading capacity of AmB increased because of the formed intermolecular interactions, such as hydrogen bonds, which also caused a lower aggregation of this drug. The assessment of in vitro toxicity against red blood cells indicated that the toxicity of AmB decreased upon encapsulation; however, its antifungal action against clinical yeasts was maintained and enhanced, as indicated by a decrease in its minimum inhibitory concentration.

## 1. Introduction

Amphotericin B (AmB) is a drug that belongs to a group of polyenic antifungals; it is also used to treat viral and parasitic diseases [1,2]. In spite of its toxicity and multiple reported side effects caused by its bioaccumulation in organs such as the liver, lungs and kidneys, AmB is considered the ‘gold standard’ in the treatment of invasive fungal infections because of its broad action spectrum, and few reports of resistant strains [3,4,5,6,7]. However, the administration of AmB is challenging because it is amphiphilic and amphoteric; it also has a very low solubility at physiological pH [6,7,8]. Nevertheless, AmB can be formulated with different methods, including colloidal dispersions stabilised by sodium deoxycholate such as Fungizone^®^. Although these formulations display the highest efficacy, they are toxic, causing side effects, such as renal failure, which is recurrent in treated patients [9]. Upon the use of liposomal formulations, some of the toxic effects are avoided, but high dose requirement, high cost and other characteristics limit their usage [10,11,12].

Although AmB, which has been used since 1959, is an antifungal, its mechanism of action is still under debate. The formation of ‘barrel type’ pores from AmB molecules embedded in a lipid membrane allows the leakage of ions and other substances vital for cell growth; as such, this mechanism is the most generally accepted proposal. Polar channels are produced via the assembly of adjacent AmB molecules with sterols and phospholipids [13]. AmB is highly active in cells that contain ergosterol and thus confers drug selectivity. However, AmB can destabilise the membranes of mammal cells that contain cholesterol, inducing toxicity. Other mechanisms have also been proposed. For instance, oxidative cell damage caused by reactive oxygen and nitrogen species generated by AmB harms cells and causes cell death [7]. In addition, sterols are sequestered by AmB aggregates located on the surface of cells (sterol sponge) [14].

The toxicity of AmB is mostly associated with its aggregates present at concentrations >1 mg/L [15]. It can be characterised through in vitro haemolysis assays, which have revealed that the toxicity of AmB has a good correlation with nephrotoxicity detected in vivo [16,17]. Nevertheless, its toxicity can be attenuated through its encapsulation in colloidal structures, such as polymer micelles (PMs) that release monomeric AmB in a controlled manner [18,19,20,21,22,23,24,25,26]. PMs can be produced spontaneously in an aqueous medium through the self-assembly of amphiphilic polymeric molecules. Therefore, they are promising vehicles because of their simple encapsulation and release mechanisms.

Despite the advantages of PMs, they present some disadvantages, such as poor stability under physiological environments and low encapsulation capacity, thereby limiting their translation to clinical applications. For example, PEG-*b*-PCL copolymers are biocompatible, but they do not have functional groups that favour chemical interactions with drugs; as such, they yield a low loading capacity (LC). These copolymers can be modified with biomolecules that enhance polymer/drug affinity without altering their biocompatibility. Early reports showed that the modification of PEG-*b*-PCL with cholesterol [27] and retinol [28] enhances their AmB loading capacity. Similarly, modification with linoleic acid and π-conjugated moieties improves the performance of this copolymer in the fabrication of micellar vehicles for curcumin [29] and doxorubicin [30], respectively. An appropriate modification of PM precursors focused on enhancing LC and affinity with the physiological target allows one to design drug vehicles that can be employed as treatment for several diseases, including cancer and neurological disorders [31,32].

In this research, PEG-*b*-PCL copolymers with two lengths of PCL segments were synthesised and modified with terminal 1,2-distearoyl-sn-glycero-3-phosphorylethanolamine (DSPE) through *N*,*N*′-dicyclohexylcarbodiimide (DCC)-activated amidation. These materials were proposed as micellar vehicles of AmB under the hypothesis that their components act synergically, thereby improving the AmB delivery system. In the presence of DSPE, a phospholipid found in cellular membranes, the loading of AmB increases as its toxicity decreases. DSPE has an amphiphilic nature. As such, it can interact hydrophobically through polyenic segments of AmB, which concomitantly prevents its aggregation. It can also act through polar interactions such as hydrogen bonds that involve polar groups on AmB and DSPE.

## 2. Materials and Methods

Methoxy-poly(ethylene glycol) (mPEG) with a molecular weight of 5.31 kDa, ε-caprolactone (CL, 98%), tin octanoate (Sn (Oct)_2_ 98%), succinic anhydride (98%), triethylamine (TEA, 99%), 4-(dimethylamino)-pyridine (DMAP, 99%), *N*-hydroxysuccinimide (NHS, 98%), *N*,*N*′-dicyclohexylcarbodiimide (DCC, 98%), 1,2-distearoyl-glycero*s-n*-3-phosphoethanolamine (DSPE, ≥99%), pyrene (Py, 98%) and other reagents and solvents used in the synthesis, purification and characterisation protocols were purchased from Sigma-Aldrich (St. Luis, MO, USA). Before the syntheses, some reagents were dried using different protocols. In particular, toluene and tetrahydrofuran were dried via distillation, and sodium and benzophenone were used as an indicator of humidity. mPEG was subjected to azeotropic distillation with dry toluene. Dioxane, dichloromethane and CL were dried with calcium hydride as a humidity adsorbent.

### 2.1. Synthesis of PEG-b-PCL Copolymers

PEG-*b*-PCL copolymers were synthesised through ring opening polymerisation under previously reported conditions [27,28]. In a typical synthesis, mPEG (5 g, 1 mmol), CL (5.3 mL, 50 mmol) and Sn (Oct)_2_ catalyst (162 µL, 0.5 mmol) were added to a round-bottom flask and dissolved in 30 mL of toluene. The reaction mixture was stirred under Ar atmosphere for 24 h at 110 °C. The reaction product was dissolved in dichloromethane (CH_2_Cl_2_) and precipitated with diethyl ether at 0 °C. Subsequently, the precipitate was filtered and dried in vacuum for 12 h at room temperature to obtain a yield of 4.77 g (85%). ^1^H-RMN (400 MHz, TMS) **PEG δ (ppm) = 3.4** (s, C**H_3_**–O–), 3.6 (s, –C**H_2_**–C**H_2_**–O–). **PCL δ (ppm) =** 1.4 (s, –C(O)–CH_2_–CH_2_–C**H_2_**–), 1.6 (q, –C**H_2_**–CH_2_–C**H_2_**–), 2.3 (t, –C(O)–C**H_2_**–), 4.0 (t, –C**H_2_**–O). Two copolymers with PCL segments with lengths of 3 and 6 kDa were synthesised and designated as PP3 and PP6, respectively.

### 2.2. Synthesis of mPEG-b-PCL-COOH

PP3 and PP6 copolymers were reacted with succinic anhydride [33]. In a typical procedure, PP3 (4 g, 0.5 mmol), succinic anhydride (0.27 g, 2.7 mmol) and DMAP (0.33 g, 2.7 mmol) were dissolved in dioxane (15.0 mL) and then added with TEA (0.3 mL, 2.2 mmol). The mixture was reacted at room temperature for 24 h under Ar atmosphere, a rotary evaporator was used to eliminate dioxane, and it was cooled by adding water. The resulting aqueous mixture was extracted with DCM (3 × 15 mL). The product was precipitated with diethyl ether at 0 °C, filtered and dried under vacuum, obtaining a yield of 78%. ^1^H-NMR (400 MHz, TMS) **PEG δ (ppm) =** 3.4 (s, C**H_3_**–O–), 3.6 (s, –CH**_2_**–CH**_2_**–O–). **PCL δ (ppm) =** 1.4 (s, –C (O) –CH_2_–CH_2_–CH**_2_**–), 1.6 (q, –CH**_2_**–CH_2_–CH**_2_**–), 2.3 (t, –C(O)–CH**_2_**-), 4.0 (t, –CH**_2_**–O). succinic ester **δ (ppm) =** 2.6 (t, C (O)–C**H_2_**–CH**_2_**–C (O)).

### 2.3. Conjugation of PCL-DSPE Copolymers

DPSE was conjugated to previously carboxylated PP3 and PP6 through amidation. In a typical procedure, PP3 (1.5 g, 0.2 mmol), DCC (0.035 g, 0.3 mmol) and NHS (0.0626 g, 0.3 mmol) were dissolved in dichloromethane (19.0 mL). Then, TEA (43 μL, 0.3 mmol) was added. The mixture was reacted for 16 h at room temperature under an inert atmosphere. Subsequently, DSPE (0.25 g, 0.34 mmol) was added, and the reaction was allowed to proceed for 24 h. The reaction mixture was evaporated with a rotary evaporator to remove DCM. The resulting product was purified by being precipitated with diethyl ether at 0 °C, and filtered and dried in vacuo to obtain **PP3-DSPE** (yield: 72.8%). ^1^H-NMR (400 MHz, TMS) **PEG δ (ppm) =** 3.4 (s, C**H_3_**–O–), 3.6 (s, –CH**_2_**–CH**_2_**–O–). **PCL δ (ppm) =** 1.4 (s, –C(O)–CH_2_–CH_2_–CH**_2_**–), 1.6 (q, –CH**_2_**–CH_2_–CH**_2_**–), 2.3 (t, –C(O)–CH**_2_**–), 4.0 (t, –CH**_2_**–O). COOH δ (ppm) = 2.6 (t, C (O)–C**H_2_**–CH**_2_**–C (O)). DSPE δ (ppm) = 0.89 (t, C**H_3_**– (CH_2_)_16_), 1.27 (m, CH_3_- (C**H_2_**)_16_), 1.8 (d, C**H_3_**–CH=CH–), 2.4 (m, –CH_2_–CH**_2_**–NH), 3.5 (q, C (O)–NH–CH**_2_**–CH_2_–O), 4.1 (NH–CH_2_–CH**_2_**–O) 4.2 (d, OC**H_2_**–CH–H**_2_**–O–), 4.7 (t, C (O) –NH–CH_2_–CH**_2_**–O), 5.5 (q, OC**H_2_**–CH–H**_2_**–OR–). A sample of PEG-DSPE taken as a reference was synthesised following a similar procedure.

### 2.4. Characterisation Techniques

Proton nuclear magnetic resonance spectra (^1^H-NMR) were obtained using a Bruker spectrometer at 400 MHz. The samples were dissolved in CDCl_3_. Chemical shifts (δ) were expressed in parts per million (ppm) with respect to a tetramethylsilane reference. The molecular weight of the mPEG initiator was evaluated through MALDI-TOF mass spectrometry by using an ultrafleXtreme mass spectrometer (Bruker), and a weight of 5.31 kDa was obtained.

The molecular weight distribution of the polymers was determined through gel permeation chromatography (GPC) by using a 5 µm 1 × 10^3^ Å Phenogel™ column in a chromatographer (Waters, Pittsburgh, PA, USA). THF was used as the mobile phase (flow rate 0.7 mL/min at 35 °C). Column calibration was performed with polystyrene (PS) standards between 1.5 and 50 kDa.

### 2.5. Critical Micelle Concentration Measurement

Critical micelle concentration (CMC) was determined in accordance with previously reported methods [34]. The dilutions of 0.05–100 mg/L copolymers containing a fixed amount of pyrene were analysed via fluorescence spectroscopy. The excitation spectra of Py from 300 to 360 nm was monitored at an emission wavelength of 390 nm by using a Cary Eclipse fluorescence spectrometer (Agilent, Santa Clara, CA, USA).

### 2.6. AmB Encapsulation

AmB-loaded PMs were prepared through a modified nanoprecipitation procedure [35]. First, an organic phase containing AmB and each copolymer was obtained by mixing a solution of AmB in methanol (1 mg/5 mL) and the corresponding copolymer in THF (1 mg/mL). The resulting solution was added to 25 mL of distilled water dropwise under constant stirring to promote the formation of dispersed nanoparticles upon the evaporation of the organic solvents. Subsequently, the suspension was centrifuged at 4400 rpm for 30 min to remove non-encapsulated AmB, and the remaining liquid phase was lyophilised. The amount of encapsulated AmB was determined as follows: each dried formulation (approximately 1 mg) was dispersed in methanol and homogenised through sonication; the volume was adjusted to 10.00 mL; and the dispersion was centrifuged. The supernatant was analysed through UV–vis to determine the amount of AmB.

### 2.7. Hydrodynamic Diameter and ζ-Potential Measurements

The hydrodynamic diameter (D_h_) of each formulation and blank particles prepared via the same procedure but without AmB was determined through dynamic light scattering (DLS). ζ-potential was measured at pH 7.0 and 25 °C by using a DIP-type cell with gold electrodes. Both measurements were performed with Nano ZS Zetasizer equipment (Marvel Panalytical, Worcestershire, UK).

### 2.8. Characterisation of AmB/PMs through DSC

The thermal properties of AmB/PMs were characterised as follows: lyophilised samples were cooled from room temperature to −60 °C at 10 °C/min. Then, they were heated to 150 °C at a rate of 10 °C/min. The analysis was carried out in a DSC 1 STAR equipment (Mettler Toledo, Colombus, OH, USA).

### 2.9. X-ray Diffraction

X-ray diffraction (XRD) experiments were performed in Xpert equipment (Marvel Panalytical, Worcestershire, United Kingdom) composed of an anode copper tube with a wavelength (λ) of 0.154069 nm. XRD was performed under the following conditions: collection time of 1 s per step, scan of 2θ = 2° to 50°, an increase of 0.1° and a detector opening of 0.5°.

### 2.10. X-ray-Induced Photoelectron Spectrometry

X-ray-induced photoelectron spectrometry (XPS) was performed to determine the composition of DSPE-conjugated copolymers and their interaction with AmB in the corresponding formulations. Measurements were made on a NAP-XPS spectrometer (SPECS Surface Nano Analysis GmbH, Berlin, Germany) equipped with a PHOIBOS 150 1-D detector and a monochromatic A1-Kα X-ray source (1486.7 eV, 13 kV and 100 W).

### 2.11. AmB Release Assessment

Approximately 1 mg of each lyophilised formulation was dispersed in 2.0 mL of distilled water and homogenised through sonication. The resulting dispersion was placed in a tube capped with a 3500 Zellutrans MWCO dialysis membrane and immersed in 15.0 mL of a release medium composed of a mixture of 1% aqueous sodium deoxycholate and DMSO in a volume ratio of 2:1. The releasing setup was maintained at 37 °C under stirring. Aliquots (1.00 mL) were withdrawn at intervals of 1, 2, 3, 6, 9, 12, 24, 36, 48, 72, 100, 120 and 144 h, and the volume of the release medium was maintained at a constant by adding a fresh medium. The amount of AmB in each aliquot was determined through UV–vis spectrometry at 415 nm. The following values were computed using Equation (1) to obtain the corresponding cumulative release (*Q*) at each time:

(1) $$Q=C_{n}V+\sum_{i=1}^{i=n-1}C_{i}V_{i},$$

where *V* is the volume of the release medium, and *C_n_* and *C_i_* are the concentrations of AmB at a given time and the former aliquots of volume *V_i_*, respectively.

### 2.12. Aggregation State

The aggregation of AmB encapsulated in PMs was assessed through UV–vis spectrophotometry. Each formulation and Fungizone^®^ were dispersed in phosphate buffer at pH 7.4 (PBS) to a final concentration of 7.0 µg/mL. A reference of monomeric AmB was obtained by dissolving AmB in methanol. The resulting dispersions were analysed using a UV–vis Evolution 300 spectrophotometer from 300 to 450 nm.

### 2.13. Haemolysis

Haemolysis was analysed in accordance with a previously published protocol [27]. Blood taken from O+ donors was added to a 1 mM EDTA solution and then centrifuged at 500× *g* for 5 min to separate red blood cells (RBC). The collected RBCs were washed twice with a phosphate buffer solution (PBS) at pH 7.4 and diluted with the same buffer until an absorbance of 0.5 AU at 540 nm was obtained. Then, 190 µL of the RBC dispersion was treated with 10 µL of each formulation dispersed in PBS. Triton X-100 and PBS were used as positive and negative controls, respectively. The treated RBC dispersions were incubated at 37 °C under continuous shaking for 1 h. Subsequently, the plate was centrifuged at 500× *g* for 5 min to separate the non-lysed cells. Haemolysis percentage was determined by measuring the absorbance of haemoglobin in a solution at 540 nm:

(2) $$Haemolysis\left(\%\right)=\frac{A_{s}-A_{NC}}{A_{PC}-A_{NC}}\times 100$$

where *A_s_* is the absorbance of drug- or polymer micelle-treated RBCs, and *A_NC_* and *A_PC_* are the absorbances of the negative and positive controls, respectively.

### 2.14. Minimum Inhibitory Concentration

An antifungal susceptibility test was performed in accordance with the microdilution in broth method of the Institute of Clinical and Laboratory Standards following the M27-A3 guidelines [36]. The dilutions of the micellar formulations and Fungizone^®^ were evaluated in the range of 0.11–15 µg of AmB/mL. Minimum inhibitory concentrations (MICs) were, visually and through turbidity measurements after 24 h, taken as the lowest drug concentration that inhibits the growth of the yeasts. The measured values were further corroborated using resazurin (7 mM) as a redox indicator. Resazurin is a cell-permeable, non-toxic blue compound that does not fluoresce. Upon entering living cells, resazurin is reduced to resorufin, a compound that is violet to pink and highly fluorescent [37].

## 3. Results and Discussion

**Scheme 1:**
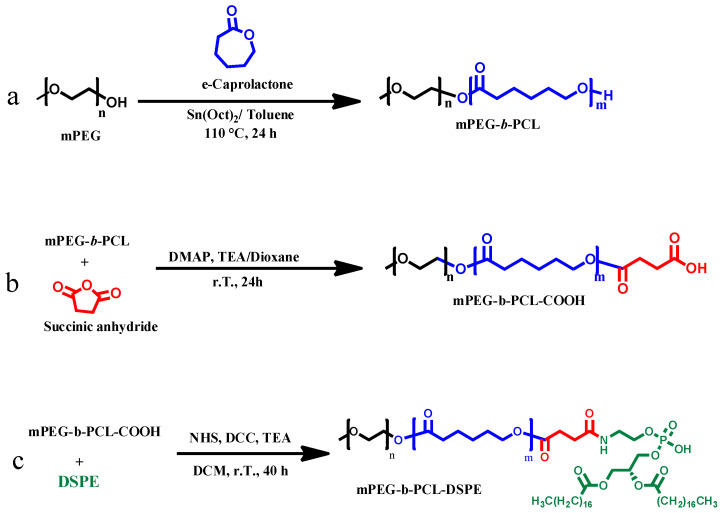
CL polymerisation starting from mPEG as an initiator via ROP (**a**), reaction of PEG-*b*-PCL with succinic anhydride (**b**) and conjugation of carboxylated copolymers with DSPE through amidation (**c**).

In this research, micellar vehicles for AmB were obtained from amphiphilic block copolymers composed of a hydrophilic segment of mPEG and a hydrophobic block of PCL terminated in DSPE. mPEG with a molecular weight of 5.3 kDa was used as the initiator for CL polymerisation via ROP (Scheme 1a). The molecular weight of the hydrophobic PCL segments was controlled on the basis of the molar ratio of CL to mPEG in the feed. The obtained copolymers were characterized via ^1^H-NMR and GPC. Figure S1a presents a representative ^1^HNMR spectrum of PP3 and shows the signals attributed to PCL and mPEG segments. The molecular weight of the PCL block in PP3 and PP6 copolymers was estimated by integrating the signal at δ = 4.0 (t, –CH**_2_**–O) of PCL and the signal at 3.6 ppm assigned to mPEG, which was taken as a reference for determining the values listed in Table 1. In addition, molecular weight dispersity indices (Ð) determined by GPC indicated that CL polymerisation was controlled in concordance with the characteristics of ROP by using Sn(oct)_2_ as a catalyst.

PP3 and PP6 were reacted with succinic anhydride (Scheme 1b) to obtain –COOH-ending copolymers. The occurrence of carboxylation at the end of PCL bocks was verified via ^1^H-NMR through the appearance of a signal at around δ (ppm) = 2.6 (t, C (O)–C**H_2_**–CH**_2_**–C (O)) that integrated for approximately four protons. This result indicated that carboxylation was complete (Figure S2).

**Figure 1:**
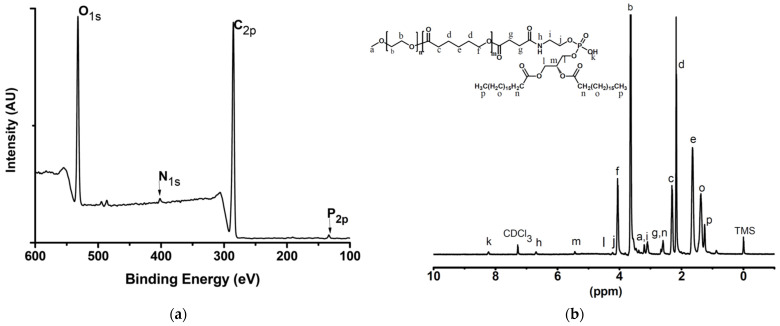
Spectral characterisation of a representative DSPE-terminated copolymer (PP3-DSPE). (**a**) XPS and (**b**) ^1^NMR spectra.

The carboxylated copolymers were further conjugated with DSPE through amidation by using DCC as a coupling agent (Scheme 1c). The elemental composition of the reaction products was corroborated through XPS. Figure 1a presents a representative spectrum of a PP3-DSPE sample. It shows signals at 532 and 287 eV attributed O and C, respectively, and signals at 400 and 134 eV corresponding to the BE of N_1s_ and P_2p_ electrons, respectively. These findings confirmed the capping of PP3 with DSPE molecules. Likewise, the ^1^HNMR spectra of the same sample revealed the resonance of protons in PEG and PCL segments and signals due to DSPE moieties, as shown in Figure 1b. Therefore, the conjugated structure was achieved.

### 3.1. CMC

**Figure 2:**
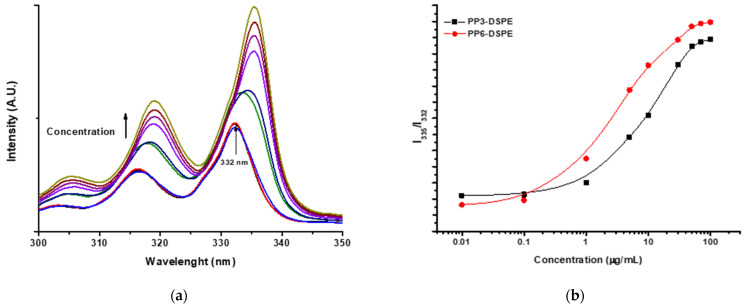
Excitation spectra of pyrene dissolved in PP3-DSPE solutions with different concentrations (**a**) and plot of the ratio of the fluorescence intensity of pyrene at 335 and 332 nm as a function of copolymer concentrations for PP3-DSPE and PP6-DSPE samples (**b**).

The CMC of the copolymers was determined through fluorescence spectroscopy by using Py as a fluorescent probe. Py is a hydrophobic compound with low solubility in water. In the presence of micellar arrangements, Py is solubilised in the lipophilic micellar nucleus, which in turn changes its optoelectronic properties [34,38], such as an increase in fluorescence intensity and a bathochromic shifting of the maxima in the excitation spectrum from 332 to 335 nm, as shown in Figure 2a for the micellization of the PP3-DSPE sample. The CMC was estimated as the inflection point in the plot of the ratio of the intensities at 332 and 335 nm (I_335_/I_332_) as a function of concentration (Figure 2b and Table 1).

The CMC was dependent on the molecular weight of the PCL segment, which was the smallest for the copolymer with the highest M_n_ (Table 1). This finding was consistent with a decrease in the solubility of the copolymer and an increase in its hydrophobicity. Upon conjugation with DSPE, CMC decreased, suggesting that dispersion forces amongst non-polar segments intensified, driving the micellization of the copolymers. A lower CMC corresponds to a more favourable self-assembly process and a higher stability of nanoparticles against dilution; this property, summed to kinetic stabilisation via the entanglement of polymer segments, allows longer circulation periods for nanoparticles and permits the controlled release of drugs [39]. The CMC of PPx-DSPE was smaller than that of the corresponding PPx precursor; it also decreased significantly compared with that of the PEG-DSPE reference widely used as a surfactant in the development of micellar drug formulations [40]. Therefore, PCL and DSPE acted synergically and enhanced the stability of the PMs against dilution.

### 3.2. Encapsulation Capacity

The capacity of PEG*-b*-PCL copolymers to encapsulate AmB was compared with the corresponding DSPE-conjugated structures (Table 2), as measured in formulations prepared via nanoprecipitation. The results showed that a short PCL segment (PP3 sample) favoured a larger loading of AmB, which could be explained by a more favourable mixing entropy. Similarly, the loading capacity significantly enhanced after the conjugation with DSPE. The obtained encapsulation order agreed with the solubilisation profiles given in Figure S2. In the evaluated range of polymer concentrations, the solubilisation of AmB was twofold in the presence of DSPE with respect to the initial PEG-*b*-PCL copolymers.

**Figure 3:**
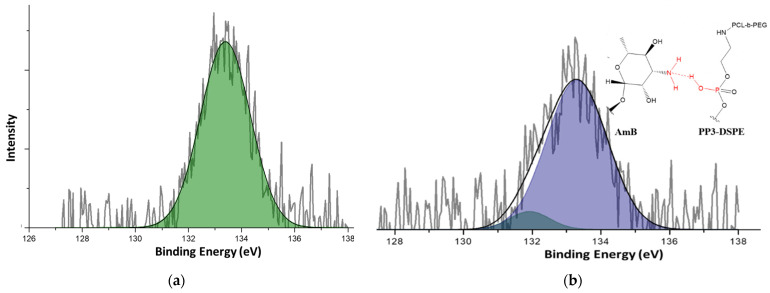
High-resolution XPS spectrum of P_2p_ in PP3-DSPE (**a**) and AmB/PP3-DSPE (**b**).

The enhanced capacity of the conjugated copolymers to encapsulate AmB suggested that the strength of intermolecular forces allowing drug encapsulation was enhanced in the presence of DSPE. Weak interactions such as Van der Wals forces were suspected, given that each DSPE moiety contains two long alkyl chains. The occurrence of intermolecular interactions that involved polar groups on the drug and the copolymer was assessed through XPS. The high-resolution spectra of phosphorus in PP3-DSPE and the corresponding AmB/PP3-DSPE nanoparticles depicted in Figure 3 were fitted to Gaussian functions. Although the signal of P_2p_ in PP3-DSPE (Figure 3a) fitted to one Gaussian peak, the same signal in AmB/PMs (Figure 3b) required two peaks, suggesting that P presented two different chemical environments. The most intense peak centred at 133 eV corresponded to the signal observed in PP3-DSPE. However, the presence of a second peak corresponding to electrons with a lower binding energy (132 eV) indicated that the electrons belonging to 2p orbitals experienced greater shielding in the presence of AmB. This increased shielding could be a consequence of the establishment of hydrogen bonding interactions between the P–OH group in the DSPE and the amino group in AmB that acted as an electron donor.

### 3.3. Particle Size and ζ-Potential Measurement

**Figure 4:**
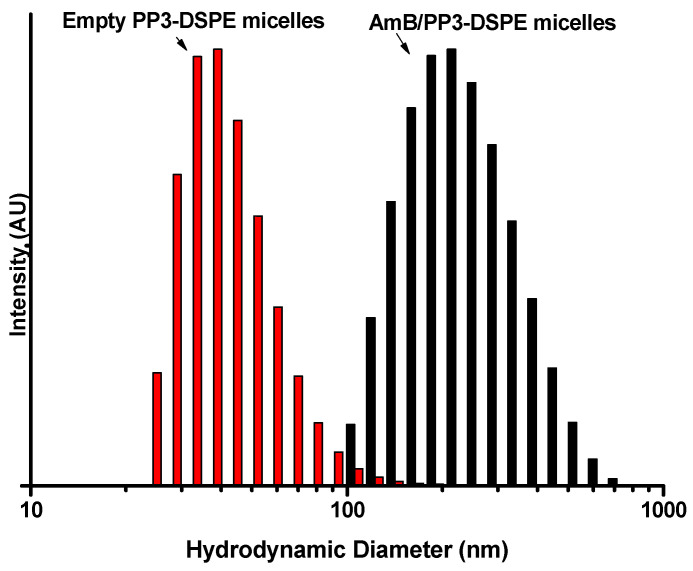
Comparison of the distribution plots of the hydrodynamic diameter of empty micelles and AmB-loaded particles obtained from PP3-DSPE.

DLS was conducted to determine the size of AmB/PMs and the corresponding empty nanocontainers. D*h* values are listed in Table 2. The empty micelles obtained from the copolymers with different PCL lengths exhibited a similar D*h* of approximately 75 nm. After their conjugation with DSPE, the diameter of the particles increased to 93 nm. By comparison, AmB/PMs exhibited diameters of about 200 nm (Figure 4). The growth of particles in the presence of AmB could suggest the formation of co-assembled structures comparable with the micellization of surfactant blends [41]. AmB, owing to its amphiphilic nature, could also have provoked the formation of micelle aggregates, as deduced from the noticeable shifting of particles size distributions to larger values. The size of the micelles was further corroborated through the AFM images of the representative samples, as displayed in Figure S3.

The ζ-potential of AmB/PMs and the empty PMs was determined by using the electrophoretic mobility technique (Table 2). The nanostructures exhibited a negatively charged surface that increased in magnitude in the presence of DSPE. The most negative values of DSPE-conjugated copolymers could be attributed to the negative charge of the phosphate group in the DSPE due to the partial dissociation of the P–OH group. This result indicated that the polar groups of DSPE were probably exposed to the water micelle interface though they were attached to PCL segments.

**Figure 5:**
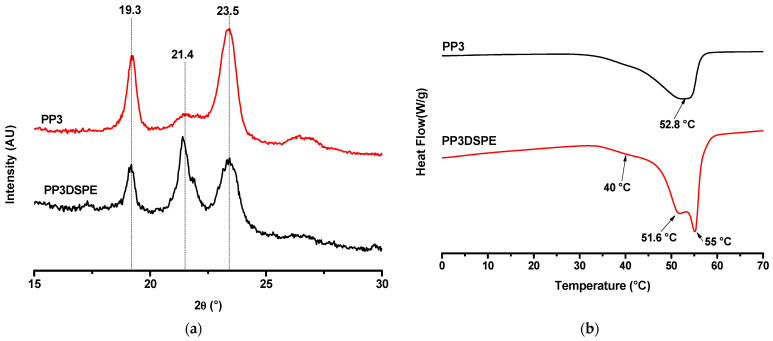
Characterisation of lyophilised formulations based on PP3 and PP3-DSPE. (**a**) DRX profiles and (**b**) DSC traces.

The structure of AmB/PM composite nanoparticles was further studied by characterising the lyophilised formulations through XRD and DSC. Figure 5a shows the diffraction profiles of the formulations obtained from PP3 and PP3-DSPE. The diffraction profile of PP3 exhibited diffraction peaks associated with PEG at 2θ of 19.3° and 23.5° [42] and a small shoulder at approximately 20° due to the crystalline nanodomains of PCL [43], which were formed upon lyophilisation. By contrast, in the profile of the nanoparticles of PP3-DSPE, the diffraction peak associated with PCL intensified. This result suggested that this substance promoted the folding of PCL blocks, inducing their crystallisation.

**Scheme 2:**
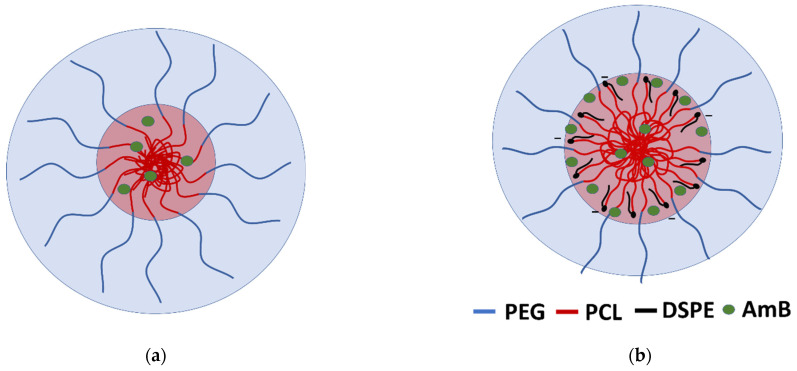
(**a**) representative structure of AmB-loaded polymer micelles composed of PEG-*b*-PCL and (**b**) PEG-b-PCL-DSPE. In raw copolymers, hydrophobic PCL segment forming the nucleus was highly amorphous allowing for AmB dissolutions. Upon conjugation, PCL crystallized and AmB encapsulation was enabled by interactions with DSPE moiety.

These two formulations were analysed through DSC (Figure 5b). The samples were cooled to −20 °C and then heated at 10 °C/min to 100 °C without any thermal erasure to obtain the traces given in Figure 5b. As such, the determined properties were found to be closely related to the morphological characteristics of the copolymers in the polymeric micelles. Although the thermogram of PP3 only showed a broad endothermic peak, mainly due to the melting of PEG crystalline domains at 52.7 °C, the nanoparticles obtained from DSPE-conjugated copolymers exhibited two peaks at 51.1 and 55.0 °C. These peaks corresponded to the domains of PCL and PEG that crystallised to a larger extent, and this observation was consistent with XRD results. The increase in PCL crystallisation in the presence of DSPE also explained the lowest CMC exhibited by the conjugated copolymers (Scheme 2).

### 3.4. Release Study

**Figure 6:**
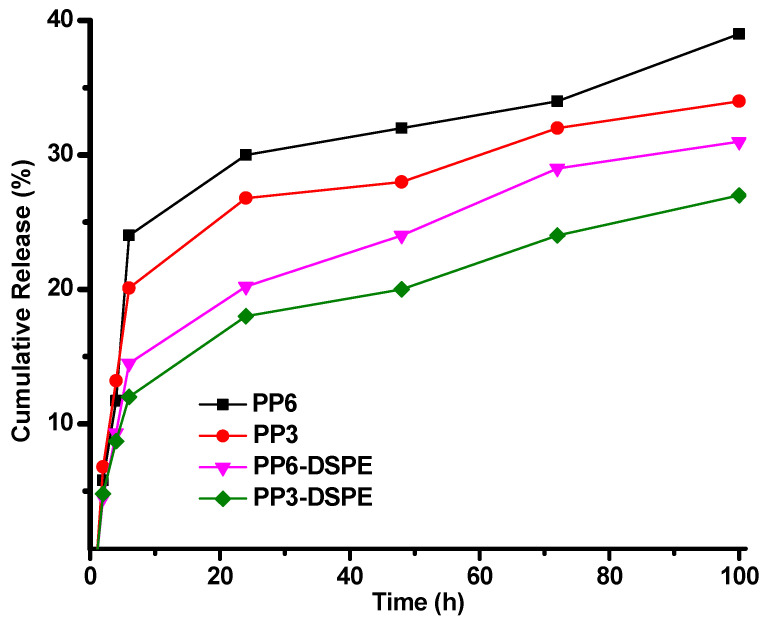
Release of AmB from micellar formulations measured for 100 h.

The release kinetics of AmB from micellar formulations was studied through dialysis. Solid formulations with a known amount of AmB were dispersed in PBS and dialysed against a medium composed of a sodium deoxycholate solution and DMSO that guaranteed sink conditions. The released AmB was monitored for 100 h by withdrawing aliquots at certain time intervals (Figure 6). The results revealed that the micellar formulations followed a bimodal release behaviour through which the occurrence of two different stages were clearly distinguished. Thus, the data corresponding to each period were separately fitted to the kinetic models used to study the release mechanisms [44,45,46,47].

Table 3 indicates that the initial stage in most of the samples fitted the Hixson Crowell and Higuchi models; this finding suggested that the release rate depended mainly on the diffusion of AmB from the particles and its dissolution in the medium [47]. Likewise, the release rate constant depended on the composition of the copolymer; that is, it decreased after conjugation with DSPE, but it was maxima for PEG-DSPE taken as a reference. First, adding DSPE to the copolymer enhanced their interaction with AmB, thereby causing an increase in the activation energy of the process. In the case of PEG-DSPE, the highest release rate was attributed to its high CMC. Therefore, the unimer exchange process was more favourable thermodynamically, enhancing the AmB dissolution. The second release stage observed after 6 h (Table S1) did not fit a particular model, indicating that multiple release mechanisms occurred when the drug was depleting.

Similarity analysis (*f*_2_) was performed to quantitatively compare the release profiles of the formulations obtained from each of the copolymers and PEG-DSPE as a reference [47]. *f*_2_ (Table S3) was computed using Equation (3), which compares the cumulative release of two different samples at a given time (*R*):

(3) $$f_{2}=50log\left\{{\left[1+\frac{1}{n}\overset{n}{\underset{t=1}{\sum }}{\left(R_{t}-T_{t}\right)}^{2}\right]}^{-0.5}\times 100\right\}.$$

The comparison of the pairs PP3/PP6, PPx/PPx-PDSE and PP3-DSPE/PP6-DSPE showed that *f*_2_ was larger than a critical value of 50, indicating that the samples did not significantly differ. However, when each of the formulations was compared with PEG-DSPE, significant differences were detected. Therefore, the release of AmB from PMs was likely controlled by the unimer exchange dynamics. In the case of the copolymers, this dynamic depended on CMC and polymer segment entanglement that delayed the dissolution of the nanostructures.

### 3.5. Haemolysis Behaviour

**Figure 7:**
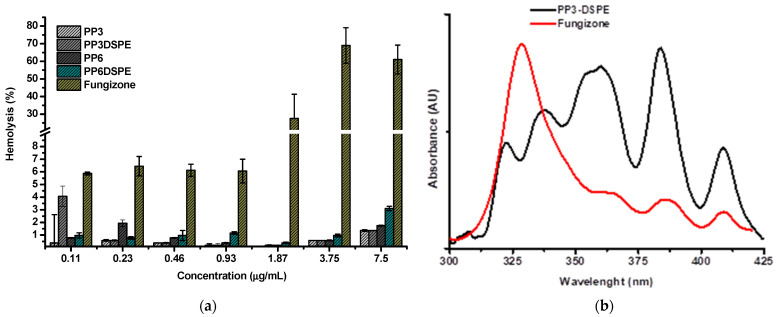
Erythrocyte toxicity of AmB-loaded PMs in comparison with commercial AmB formulation (**a**). (**b**) Aggregation state of AmB in Fungizone ^®^ and a micellar formulation obtained using PP3-DSPE as a polymeric precursor.

The haemolytic behaviour of AmB encapsulated in PMs was compared with that of Fungizone ^®^ at different AmB concentrations in the range of 0.11–7.5 µg/mL (Figure 7a). At concentrations below 1.87 μg/mL, the haemolysis of the micellar formulations and Fungizone^®^ was lower than 5%. However, as the concentration of AmB increased, Fungizone^®^ became more cytotoxic. The dependence of the haemolytic effect of Fungizone^®^ with concentration correlated with the presence of aggregates, which spontaneously formed when the concentration of AmB exceeded 1 μg/mL.

UV–vis spectroscopy provided reliable information about the aggregation state of AmB in the aqueous medium, given that the monomers (non-aggregated form) and the aggregates presented distinctive absorptions. In Figure 7b, the spectra of Fungizone^®^ and a representative micellar formulation (AmB/PP3-DSPE) exhibited λ_max_ at 408, 385 and 365 nm because of AmB monomers and an absorption band at 345 nm assigned to the aggregates [48]. Although the most intense absorption of Fungizone^®^ corresponded to the aggregates, the spectrum of the micellar formulations was dominated by the absorption of monomers. This trend agreed with a previous study, which indicated the toxicity of AmB in the presence of aggregates [49]. Thus, an alternative to obtain safer formulations of AmB could be suppressing its self-assembly and controlling its release [50].

### 3.6. MIC

The antifungal action of AmB formulations was defined as the MIC and evaluated against clinical isolates (Table 4). According to the provided values, the isolates were mostly sensitive to Fungizone except *C. auris* reference 537-PUJ-HUSI, which had a MIC of 3.35 μg/mL. AmB encapsulated in PMs had lower MICs, indicating that it had an improved efficacy compared with that of Fungizone^®^. The action spectrum of the former also broadened, as shown by a reduction in the MIC of the resistant strain to show values comparable with the sensitive isolates. The micellar formulations obtained from the copolymers with the lowest molecular weight presented the highest efficiency. In the PP3 series, DSPE-conjugated nanoparticles did not show an improvement. They contrasted with the PP6 series where in the presence of the phospholipid the MICs decreased.

The improved performance of micellar formulations compared with that of Fungizone^®^ could be attributed to the continuous feeding of AmB provided by the polymeric particles to the medium; consequently, premature drug inactivation caused by its interaction with proteins is avoided [51]. Fungizone^®^ is present in AmB dispersed with sodium deoxycholate; as such, when it is dissolved in the medium, a significant fraction of the drug is wasted.

## 4. Conclusions

Copolymers composed of polyethyleneglycol and poly(ε-caprolactone) are promising precursors of micellar nanocarriers for AmB; however, the absence of functional groups leads to a low encapsulation capacity, reducing its potential in clinical applications. When PEG-*b*-PCL is terminally modified with DSPE through DCC-activated amidation, the stability of the resulting materials against dilution was higher, as indicated by a decrease in CMC with respect to the initial copolymers. This modification also increased their AmB loading capacity to approximately 16%, thereby generating colloidal particles with hydrodynamic diameters of approximately 200 nm. The haemolysis of erythrocytes decreased, indicating that the encapsulation of AmB in PMs produced formulations that were less toxic than Fungizone^®^. The MIC decreased compared with that of Fungizone^®^. Therefore, micellar formulations exhibited a controlled release of AmB and enhanced the performance of AmB against the tested strains.

## Figures and Tables

**Table 1:** Average composition, molecular weight, Đ and CMC.

Sample	Average Composition	Mn (kDa)	Đ	CMC (μg/mL)
PEG	m(PEG)_121_	5.3 *	1.1	
PP6	m(PEG)_121_-(PCL)_75_	13.8	1.2	0.66
PP3	m(PEG)_121_-(PCL)_26_	8.2	1.2	1.4
PEG-DSPE	m(PEG)_121_-DSPE	5.3	1.1	8.5
PP6-DSPE	m(PEG)_121_-(PCL)_75_-DSPE	14.5	1.2	0.30
PP3-DSPE	m(PEG)_121_-(PCL)_26_-DSPE	9.0	1.2	1.1

M_n_ was estimated via ^1^HNMR. * Determined through MALDI-TOF.

**Table 2:** Characterisation of micellar formulations. Drug content, hydrodynamic diameter (Dh) and ζ-potential values of empty and AmB-loaded nanoparticles measured at pH 7.0.

Sample	AmB Loading (%wt.)	Empty PMs		AmB/PMs	
		Dh nm (PDI)	ζ-Potential (σ) mv	Dh nm (PDI)	ζ-Potential (σ) mv
PP6	8.37 ± 0.34	75 (0.41)	−5.8 (0.65)	198 (0.27)	−4.2 (0.9)
PP3	10.62 ± 0.78	74 (0.43)	−5.4 (0.39)	206 (0.33)	−3.0 (1.3)
PP6-DSPE	15.29 ± 0.34	93 (0.40)	−18.0 (0.72)	217 (0.26)	−7.5 (1.9)
PP3-DSPE	16.40 ± 0.18	93 (0.44)	−13.3 (0.73)	226 (0.25)	−8.8 (0.1)
PEG-DSPE		84 (0.26)	−12.7 (0.67)	149 (0.24)	−10.4 (1.6)

**Table 3:** Kinetic analysis of the release models.

Model	Parameter	PP6	PP3	PP6-DSPE	PP3-DSPE	PEG-DSPE
		0–6 h				
Orden 0	R^2^	0.978	0.979	0.987	0.952	0.988
	K_o_	4.570	3.833	2.789	2.265	5.227
Orden 1	R^2^	0.664	0.621	0.632	0.603	0.635
	k	1.026	0.957	0.976	0.897	0.966
Korsmeyer–Peppas	R^2^	0.843	0.814	0.823	0.799	0.825
	k_KP_	1.895	1.858	1.922	1.888	1.818
	N	3.250	3.080	3.130	2.905	3.094
Higuchi	R^2^	0.952	0.994	0.996	0.986	0.995
	k_H_	0.157	0.134	0.097	0.080	0.182
Hixson Crowell	R^2^	0.973	0.984	0.990	0.957	0.992
	k_HC_	0.017	0.0138	0.0098	0.0079	0.0194
Baker–Lonsdale	R^2^	0.913	0.842	0.874	0.814	0.853
	k_BL_	0.0601	0.0546	0.0476	0.0421	0.0625

**Table 4:** MIC (µg/mL) of AmB-loaded copolymers and commercial formulation.

Strain	Reference	Fungizone^®^	PP6	PP3	PP6-DSPE	PP3-DSPE
*C. albicans*	SC5314	0.46	0.23	0.11	0.23	0.11
*C. glabrata*	ATCC 2001	0.93	0.46	0.11	0.23	0.11
*C. auris*	435-PUJ-HUSI	0.93	0.93	0.23	0.23	0.23
*C. auris*	537-PUJ-HUSI	3.75	0.93	0.23	0.93	0.23

## Data Availability

The data presented in this study are available on request from the corresponding author.

## Supplementary Materials

The following are available online at https://www.mdpi.com/article/10.3390/polym13111747/s1. Table S1: fitting parameters of the second release stage. Table S2: similarity analysis. Figure S1: ^1^H-NMR spectra of (a) PP3 and (b) PP3-succinic acid copolymers. Figure S2. solubility profiles of AmB in the presence of copolymers. Figure S3. AFM images of representative samples AmB/PP3 (a) and AmB/PP3-DSPE (b).
