# Supplementary material for: Phospholipid-Conjugated PEG-b-PCL Copolymers as Precursors of Micellar Vehicles for Amphotericin B
Source: Polymers (Basel). 2021 May 27;13(11):1747. doi: 10.3390/polym13111747 (PMC8199447; doi:10.3390/polym13111747)
Supplement: Supplementary file 1 [file polymers-13-01747-s001.zip › polymers-1195020-supplementary.pdf]

polymers

MDPI

Supplementary Materials

Phospholipid-conjugated PEG-b-PCL copolymers as precursors of micellar vehicles for Amphotericin B

Elsa R. Arias, Angie Angarita-Villamizar, Yolima Baena, Claudia Parra-Giraldo and Leon D. Perez

Table 1. Fitting parameters for the second release stage.

Model	Parameter	PP6	PP3	PP6-DSPE	PP3-DSPE	PEG-DSPE
		24 h – 100 h				
**Orden 0**	R^2	0,957	0,959	0,964	0,986	0,962
	Ko	0,116	0,101	0,147	0,123	0,299
**Orden 1**	R^2	0,971	0,958	0,951	0,986	0,938
	k	0,003	0,003	0,006	0,006	0,006
**Korsmeyer-Peppas**	R^2	0,873	0,902	0,981	0,943	0,995
	k_{KP}	0,570	0,636	0,956	0,977	0,743
	**n**	0,170	0,172	0,312	0,287	0,345
**Higuchi**	R^2	0,910	0,938	0,980	0,966	0,989
	k_H	0,017	0,015	0,022	0,018	0,045
**Hixson Crowell**	R^2	0,952	0,959	0,966	0,986	0,974
	k_{HC}	0,0005	0,0004	0,0006	0,0005	0,0016
**Baker-Lonsdale**	R^2	0,972	0,958	0,952	0,986	0,923
	k_{BL}	0,0005	0,0005	0,0008	0,0008	0,0008

Table 2. Similarity analysis.

**Formulation pair**	**f_2 parameter**
PP6 and PP3	74,8
PP6 and PP6-DSPE	58,8
PP3 and PP6-DSPE	69,1
PP3 and PP3-DSPE	58,9
PP6-DSPE and PP3-DSPE	75,5
PP6-DSPE and PEG-DSPE	37,9
PP3-DSPE and PEG-DSPE	34,6

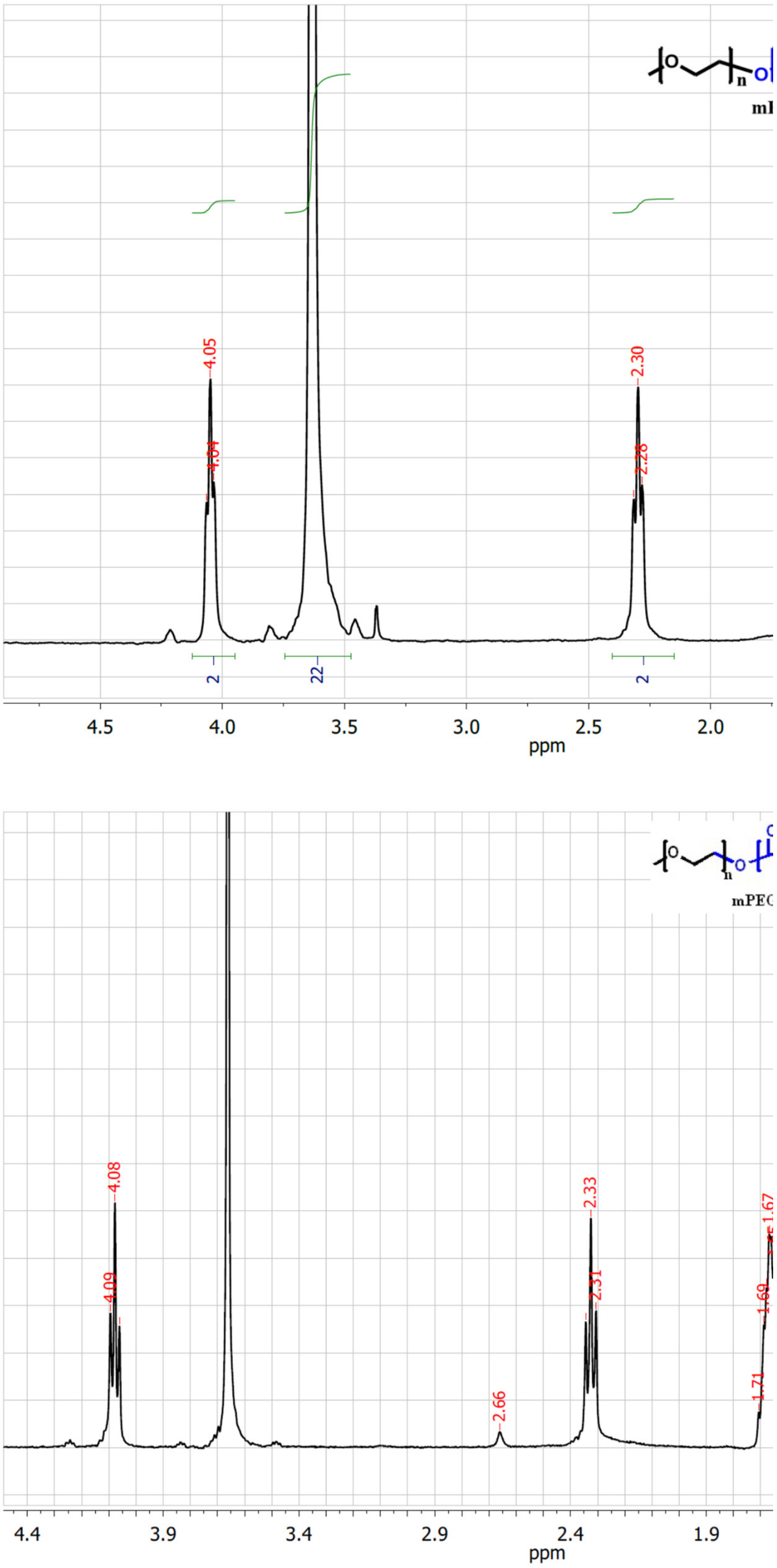

Figure S1. 1H-NMR spectra of (a) PP3 and (b) PP3-Succinic acid copolymers.

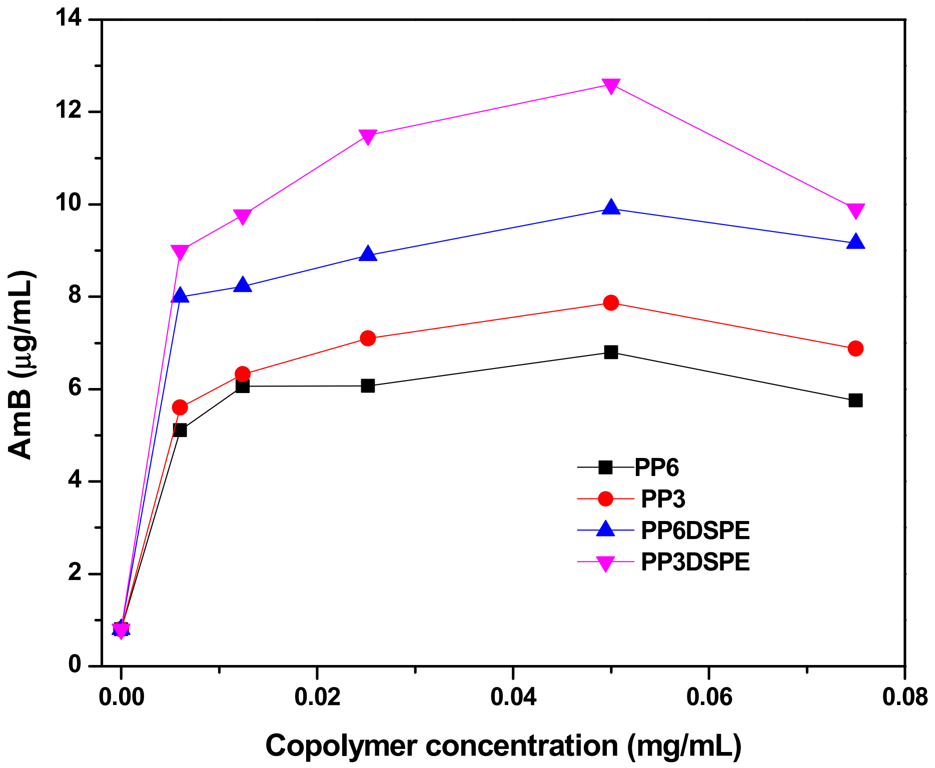

Figure S2. Solubility profiles of AmB in the presence of copolymers.

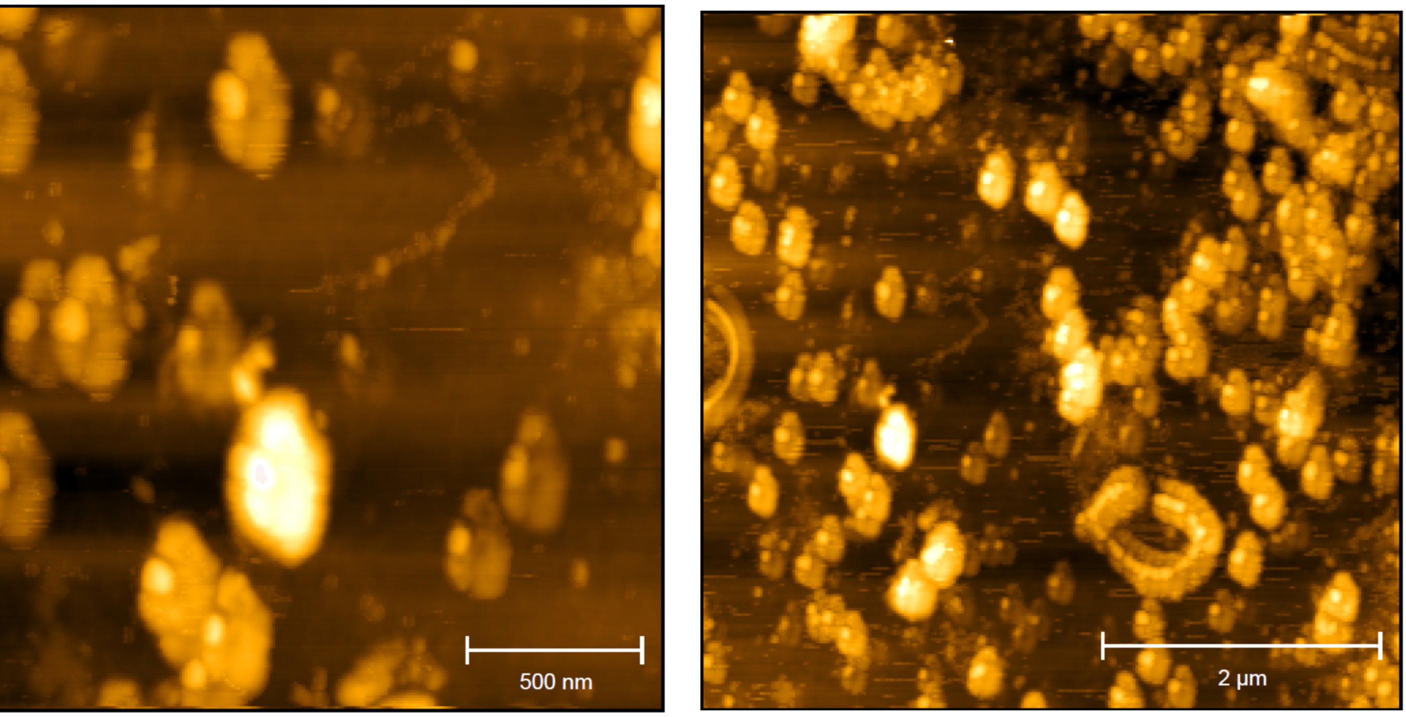

(a) (b)

Figure S3. AFM images for representative samples AmB/PP3 (a) and AmB/PP3-DSPE (b).
